# Supplementary material for: Identification of photoperiod-induced specific miRNAs in the adrenal glands of Sunite sheep (Ovis aries)
Source: Front Vet Sci. 2022 Jul 22;9:888207. doi: 10.3389/fvets.2022.888207 (PMC9354845; doi:10.3389/fvets.2022.888207)
Supplement: Supplementary file 1 [file Data_Sheet_1.ZIP › Supplementary file/miRDeep2 file.zip!/miRDeep2 file/Novel_Structure/SP42A3/Novel_142.pdf]

Provisional ID	:	NC_019460.2_8532
Score total	:	23.2
Score for star read(s)	:	3.9
Score for read counts	:	16.4
Score for mfe	:	1.2
Score for randfold	:	1.6
Score for cons. seed	:	
Total read count	:	44
Mature read count	:	42
Loop read count	:	0
Star read count	:	2

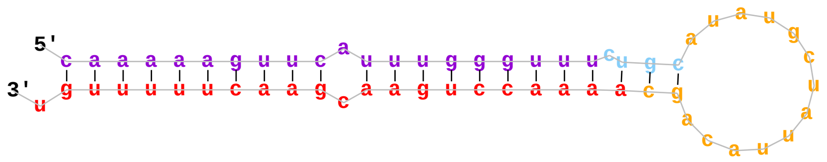

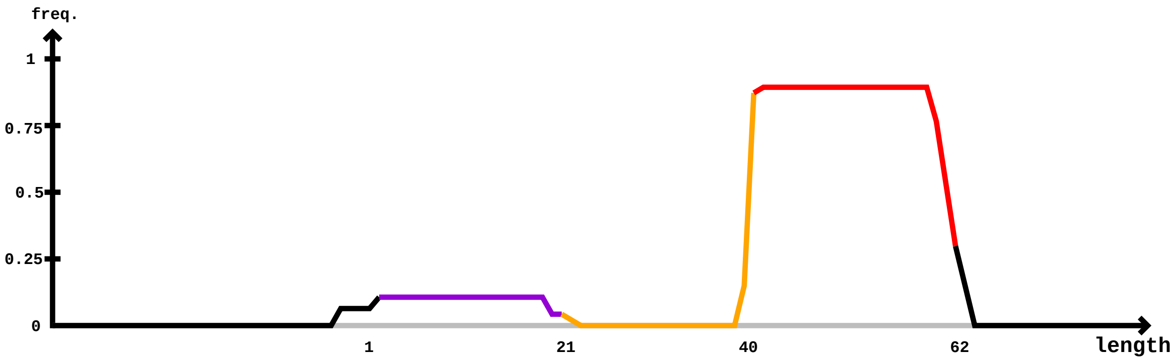

Star Mature

```
5'- aauuccacaagcaaugauugaauugguuuggccaaaaaguucauuuggguuucugcauaugcuauuacagcaaaaccugaacgaacuuuuuguccaacccaauagaugcaaa  -3'   obs
    aauuccacaagcaaugauugaauugguuuggccaaaaaguucauuuggguuucugcauaugcuauuacagcaaaaccugaacgaacuuuuuguccaacccaauagaugcaaa         exp
    ..............((..(..(((((.((((.((((((((((.(((((((((.(((.............)))))))))))).)))))))))).)))).)))))..)..))..   reads   mm   sample
    ............................uggccaaaaagCucauuugggu..............................................................   2       1    seq
    ............................uggccaaaaaguucauuugAgu..............................................................   1       1    seq
    ................................caaaaaguucGuuuggguuu............................................................   2       1    seq
    ......................................................................Aaaaaccugaacgaacuuuu......................   4       1    seq
    ......................................................................caaaaccugaacgaacuuuuu.....................   1       0    seq
    ......................................................................caaaaccugaacgaacuuuuug....................   2       0    seq
    .......................................................................aaaaccugaacUaacuuuu......................   2       1    seq
    .......................................................................aaaaccAgaacgaacuuuuu.....................   9       1    seq
    .......................................................................aaaaccugaacAaacuuuuug....................   3       1    seq
    .......................................................................aaaaccAgaacgaacuuuuug....................   6       1    seq
    .......................................................................aaaaccugaacAaacuuuuugu...................   1       1    seq
    .......................................................................aaaaccugaacgaacuuuuugC...................   3       1    seq
    .......................................................................aaaaccugaaUgaacuuuuugu...................   10      1    seq
    ........................................................................aaGccugaacgaacuuuuu.....................   1       1    seq
```
